# Status of rpoB gene mutation associated with rifampicin-resistant *Mycobacterium tuberculosis* isolated in a rural setting in Nepal

**DOI:** 10.1099/acmi.0.000202

**Published:** 2021-02-11

**Authors:** Saroj Adhikari, Bhuvan Saud, Sunil Sunar, Sheshraj Ghimire, Bhawani Prasad Yadav

**Affiliations:** ^1^​ Pyuthan District Hospital, Pyuthan, Nepal; ^2^​ Department of Medical Laboratory Technology, Janamaitri Foundation Institute of Health Sciences, Hattiban, Lalitpur, Nepal

**Keywords:** *Mycobacterium tuberculosis*, GeneXpert, rifampin resistance, rpoB gene, rural setting, Nepal

## Abstract

*
Mycobacterium tuberculosis* ranks among the top 10 causes of deaths in Nepal despite the country having a long history of national tuberculosis prevention programmes that have proved very successful in the control of tuberculosis. Several cases of active or latent tuberculosis are still missing despite that the number of infected individuals is increasing each year. Microscopy has its own limitations and factors like low bacterial load, quality of sample, quality of smear, experience of microscopist etc. influence the overall sensitivity of the test. The implementation of a molecular technique-based rapid, point-of-care testing system offers higher sensitivity in the early diagnosis of tuberculosis. Cepheid GeneXpert is the most commonly used molecular technology in Nepal. It is a cartridge-based semi-quantitative, nested real-time PCR-based diagnostic system. It detects mutations in the beta-subunit of RNA polymerase (rpoB) gene that lead to rifampicin resistance (RR) in *M. tuberculosis* complex. The present study aims to increase our understanding of the epidemiology of mutations in the rpoB gene in tuberculosis-positive patients by using the Xpert MTB/RIF assay in a rural setting in Pyuthan Hospital, Nepal. Sputum from 2733 patients was tested for the diagnosis of tuberculosis using the Cepheid GeneXpert system between July 2018 and January 2020 at Pyuthan Hospital. Two hundred and ninety-seven of these samples (10.86 %) were positive for *M. tuberculosis *, of which 3.3 % (10/297) were rifampicin-resistant. Among rifampicin-resistant tuberculosis (RR-TB) patients, 50.0 % (5/10) showed mutations located in codons 529–533 (probe E) of the rpoB gene, followed by others. The GeneXpert system can be a convenient, highly sensitive, rapid and accurate tool for the diagnosis of tuberculosis, also identifying RR-TB and at the same time determining the molecular epidemiology of rifampin resistance-associated mutations in rural and/or resource-limited laboratory settings.

## Introduction

Tuberculosis (TB), a preventable and curable illness, is caused by *Mycobacterium tuberculosis* and other mycobacteria belonging to *M. tuberculosis* complex. The lungs are the most commonly affected site, but it can affect any other part of the human body. According to the World Health Organization (WHO), in 2018, an estimated 10 million people around the world were affected by tuberculosis, of whom 57 % were male, 32 % female and 11 % children. About one-quarter of the world’s population has latent TB and 5–15 % of them have a lifetime risk of falling ill due to infection. The risk increases with malnutrition (3×), immune impairment, human immunodeficiency virus/acquired immunodeficiency syndrome illness (19×), tobacco use (1.6×) and alcohol use (3.3×) [1].

Nepal is landlocked by two TB-epidemic countries, India and PR China [2]. Worldwide, the burden of TB is highest in India, followed by other countries such as PR China, Indonesia, the Philippines, Pakistan, Nigeria, Bangladesh and South Africa [1]. According to the National Tuberculosis Center (NTC), the incidence of tuberculosis is increasing in Nepal. In the fiscal year 2015–16, 32 056 new tuberculosis cases were detected, while there were 31 764 cases in 2016–17 and 32 474 cases in 2017–18 [3]. TB ranks seventh among the leading cause of deaths in Nepal and around 6000 people died of tuberculosis in the fiscal year 2017–18; this means that every day an estimated 87 people became ill from tuberculosis and 16 people died [3, 4]. According to WHO estimates for Nepal, in 2018, for every 100 000 people the total TB incidence rate was 151 (133–170), the multidrug-resistant (MDR)/RR-TB incidence rate was 5 (2.8–8), the HIV-negative TB mortality rate was 19 (13-26) and the HIV-positive mortality rate was 0.33 (0.24–0.42).

MDR tuberculosis has become a major challenge for health security in Nepal. The estimated proportion of TB cases with MDR/RR-TB in 2018 was 2.2 % (1.1–3.6) in new cases and 15 % (9.6–22) among previously treated cases [5]. Nepal has committed to the WHO’s End TB Strategy, which has set milestones for 2020, 2025 and 2030, and a target for 2035, keeping various indicators for 2015 as baseline [3], but poverty and malnutrition aggravate the burden of TB in Nepal.

After WHO recommendations in 2010, use of the rapid molecular test device Gene Xpert MTB/RIF has increased substantially, which has helped greatly in reaching the milestones. Because the test detects TB infection within 2 h and also detects rifampicin resistance simultaneously, the Gene Xpert MTB/RIF also addresses the socio-economic target in the End TB Strategy. People who are diagnosed with RR can be enrolled for a second-line regimen by the clinicians on the day of diagnosis. The Gene Xpert MTB/RIF was installed at Pyuthan Hospital in August 2017, and since then it has served the people of Pyuthan uninterruptedly to date. The aim of present study is to understand the rifampicin resistance pattern in the Pyuthan district using the Xpert MTB/RIF test.

## Methods

### Study site and sample collection

Pyuthan District lies in the Rapti Zone in Province 5 of Nepal. Pyuthan Khalanga is the district’s headquarters. The district, with an area of 1309 km^2^, comprises two rural municipalities and seven village bodies. Retrospective demographic and laboratory data for the patients were obtained from hospital records and the pathology laboratory of the hospital. A total of 2733 sputum samples were tested for the diagnosis of tuberculosis using the Cepheid Gene Xpert system at Pyuthan Hospital from 2018 to 2019. Samples are received in the hospital’s pathology laboratory from the Out-Patient Department (OPD) and the In-Patient Department (IPD) and through contact tracing from remote areas of the district by courier (in cold chain) and by drones. Approximately 2–4 ml of sputum sample is collected under the direct supervision of technicians or other healthcare personnel. Unacceptable samples are disposed of and resampling is requested.

### Sample processing

Samples were tested using the Cepheid GeneXpert system. Each sample was assigned a unique laboratory number for identification. An approximately double volume of sample reagent was added to each sample contained in the centrifuge tube (2 : 1 dilution, sample reagent : sputum), and the tubes were screw-capped tightly and vigorously shaken 10–20 times and allowed to incubate for 10 min. Again, each sample was shaken vigorously and further incubated at room temperature for 5 min. Two mililitres of liquefied sample was transferred to the sample chamber of the Xpert MTB/RIF cartridge with the help of a disposable transfer pipette provided with each cartridge. The cartridge was then placed into one of the modules of the GeneXpert system, which automatically performed the test. The machine is regularly calibrated with the help of calibrator.

The GeneXpert MTB/RIF uses molecular beacons in five overlapping regions of the rpoB gene. The probes are able to detect mutations in the codons 507 to 511 (probe A), 511 to 518 (probe B), 518 to 523 (probe C), 523 to 529 (probe D) and 529 to 533 (probe E). Data for mutations in various regions of the rpoB gene were obtained from individual RR-TB patients’ detailed results from the Cepheid GeneXpert system. If the target MTB sequence is detected and any of the six overlapping probes, i.e. probe A, probe B, probe C, probe D and probe E, show a mutation that falls within the valid delta-cycle threshold setting, the test result is flagged as ‘rifampicin resistant’ by the machine, but if the signal is not sufficient then the test result is flagged as ‘rifampicin indeterminate’.

## Results

Two thousand seven hundred and thirty-three samples were tested for tuberculosis, with 297 (10.87 %) testing positive. Of the positive patients, 205 (69.03 %) were male and 92 (30.97 %) were female (Table 1). In the case of rifampicin sensitivity, 285 (95.96 %) patients were sensitive, 10 (3.36 %) were resistant and 2 (0.68 %) were indeterminate. All of the patients showing rifampicin resistance were male (Table 2). In the age-wise distribution of all positive cases, 155 (52.18 %) patients were aged 55 and above. Among positive male patients, 108 (52.68 %) were aged 55 years or above, followed by 64 (31.22 %) from the age group 35–54 years, 32 (15.6 %) from the age group 15–34 years and 1 from the age group 0–14 years. Among positive female patients 47 (51.087 %) from the age group 55 years and above, followed by 26 (28.261 %) from the age group 15–34 years, 17 (18.478 %) from the age group 35–54 years and 2 (2.174 %) from the age group 0–14 years (Table 3). In ethnicity-based classification among positive patients, 142 (47.81 %) were Janajati, followed by 75 (25.25 %) Dalits, 68 (22.9 %) Chhetri and 12 (4.04 %) Brahmin (Table 4). Among rifampicin-resistant isolates, five (50 %) showed a mutation located in the region of probe E of the rpoB gene, followed by 2 (20 %) in the probe D region, 2 (20 %) in probe C and 1 (10 %) in probe B. No mutation was observed in the probe A region (Table 5).

**Table 1.** Sex-wise positivity rate for *M. tuberculosis
*

Serial Number	Gender	Positive (*n*=297)	Percentage
1	Male	205	69.03 %
2	Female	92	30.97 %

**Table 2.** Rifampicin sensitivity results for *M. tuberculosis* positive cases

Serial Number	Rifampicin sensitivity (*n*=297)					
	Sensitive		Resistant		Indeterminate	
	no.	%	no.	%	no.	Percentage
1.	285	95.96	10 (All male)	3.36	02	0.68%

**Table 3.** Age wise distribution of *M. tuberculosis* positive patients

Serial Number	Age group (in years)	Positive patients			
		Male (*n*=205)		Female (*n*=92)	
		no.	Percentage	no.	Percentage
1	0–14	01	0.5 %	02	2.17 %
2	15–34	32	15.6 %	26	28.261 %
3	35–54	64	31.22 %	17	18.478 %
4	55 or above	108	52.68 %	47	51.087 %

**Table 4.** Ethnicity-based classification of *M. tuberculosis* positive cases

Serial Number	Ethnicity	no. (*n*=297)	Percentage
1	Janajati	142	47.81
2	Dalits	75	25.25
3	Chhetri	68	22.9
4	Brahmin	12	4.04

**Table 5.** RR-TB cases that showed mutation at various regions of the beta-subunit of the RNA polymerase (rpoB) gene that leads to rifampicin resistance in *M. tuberculosis
*

Serial Number	Location of mutation	Probe	no. (*n*=10)	Percentage
1	Codon 507 to 511	Probe A	00	0 %
2	Codon 511 to 518	Probe B	01	10 %
3	Codon 518 to 523	Probe C	02	20 %
4	Codon 523 to 529	Probe D	02	20 %
5	Codon 529 to 533	Probe E	05	50 %

## Discussion

Tuberculosis has a high morbidity rate in developing countries and remains a major public health challenge in Nepal. For the diagnosis of TB, there are 604 microscopic centres and 56 Gene Xpert centres in Nepal. However, culture and drug susceptibility testing facilities for DR-TB patients are limited to two national reference laboratories at the central level. In 2017/18, 32 474 cases of TB were notified and registered, among which 98 % (31 723) were incident TB cases. The burden of TB was high in Province 2, Province 3 and Province 5, which together reported 66 % of all cases. Province 3 had the highest incidence, with 24 % of the national total [3]. In this study, we found a positivity rate of 10.86 % among all successfully tested samples. A study from India showed that 60.41 % of samples from rural areas were positive for *M. tuberculosis* [6]. In our study, he lower positivity rates may have been due to lack of proper patient selection (those with typical symptoms of TB) during contact tracing by less trained professionals. However, the positivity rate was much higher in samples sent from OPD or IPD of the hospital. More than 52 % of the total positive patients were from the age group 55 years or above. More than 70% of the positive patients were from the socio-economically marginalized group and those who live a life of hardship around the highlands of the valley. Malnutrition, old age or lack of awareness may increase the incidence of this life-threatening disease. This indicates that to combat tuberculosis, reduction of poverty, proper nutrition and improvements to the lifestyle of the elderly have important roles to play.

In our study, rifampicin resistance was found in 3.3 % of patients. The rifampicin resistance rate is lower than that in studies conducted in Yenagoa, Nigeria (14.7 %) [7]; India (9.2 %) [6]; Gaborone, Botswana (8.0 %) [8]; and Ethiopia (4.9 %) [9]. RR-TB patients with a mutation located in codons 529 to 531 (probe E) of the rpoB gene accounted for 50 %, followed by 20 % in codons 523 to 529 (probe D), 20 % in codons 523 to 529 (probe C) and 10 % in codons 511 to 518 (probe B). No mutation was observed in codons 507 to 511. A study conducted in Nepal by Poudel *et al.* found mutations in codon 531 (58.7 %), codon 526 (15.6 %) and codon 516 (15.6 %) [10], and Makadia *et al.* from India found that out of 30 RR-TB patients, 53.33 % showed a mutation in codon 531, 16.66 % in codon 526 and 10 % in codon 516 [11]. Mani *et al.* found mutations in codon 531 (53 %) and codon 526 (19 %) among RR-TB patients in many states of India [12]. The mutations observed in our study are similar to those in India. According to the 2011 Nepal census, 36 858 people were ‘absentee population’ in the district. In the district, 32.2 % people live below the poverty line, with many people leaving for India for employment. The similarity in the circulating strains may be attributed to large numbers of poor people from the district migrating to India for employment.

Determination of RR-TB is of great significance in determining MDR TB, as 90 % of all RR-TB patients are also resistant to isoniazid [13]. Survey data in Nepal suggested that the prevalence of MDR-TB was 2.2 for new cases and 15.4 % among retreatment cases. In the fiscal year 2016–17 a total of 257 RR/MDR TB, 91 pre-XDR TB and 18 XDR TB were found in Nepal. Approximately 42.3 % of MDR patients may require pre-XDR treatment, while 4 % may require XDR treatment [14]. The unavailability of early screening of presumptive TB cases and rapid drug sensitivity testing facilities, and the remoteness of many the parts of the country are the biggest challenges in combating and controlling the spread of DR/MDR tuberculosis.

The Tuberculosis Control Program in Nepal is one of best implemented tuberculosis control programmes in the world. The directly observed treatment short-course programmes have a treatment success rate of 85 % in Nepal [15]. Even in the Pyuthan District every aspect of the tuberculosis control programme is being implemented successfully. Despite this, it is believed that there are many more cases still missing in the district. This might be due to lack of timely access to the hospital due to the harsh geography of the hills, non-compliant patients, social stigma associated with the disease, insufficiently sophisticated laboratories for testing drug-sensitivity to tuberculosis or inadequate testing of the at-risk population. With the introduction of the Gene Xpert system, it has become possible to identify tuberculosis and at the same time determine RR/MDR-TB cases that would have been missed by microscopy.

## Conclusion

The installation of the GeneXpert system has made a great contribution to combating tuberculosis. High sensitivity comparable with that of culture, higher specificity, quick report production and simultaneous determination of whether the tuberculosis-positive case is MDR are the features that makes it a valuable tool in addressing the set targets of the End TB Strategy. Although the unit cost for tuberculosis tests using the Gene Xpert system is a little high, the positive impacts it has on health and the economy offset its cost.

Early diagnosis is helping in the control of the spread of the disease through droplets. The data obtained for mutation among RR-TB cases can significantly enhance our understanding of the epidemiology of mutation. This can help in monitoring the types in circulation and aid in determining the cause of shifts in mutation trends. There is an urgent need for the installation of more such systems to efficiently combat tuberculosis in Nepal, as also suggested by the country’s National TB Prevalence Survey 2018–19.

### Data Availability

The data used to support the findings of this study are available from the corresponding author upon request.
